# Large-aperture refractive lenses for momentum-resolved spectroscopy with hard X-rays

**DOI:** 10.1107/S0909049513011722

**Published:** 2013-05-18

**Authors:** Hiroshi Fukui, Markus Simon, Vladimir Nazmov, Jürgen Mohr, Kenneth Evans-Lutterodt, Aaron Stein, Alfred Q. R. Baron

**Affiliations:** a Materials Dynamics Laboratory, RIKEN SPring-8 Center, 1-1-1 Kouto, Sayo, Hyogo 679-5148, Japan; b Karlsruhe Institute for Technology, Institute for Microstructure Technology, Kaiserstrasse 12, 76131 Karlsruhe, Germany; c Brookhaven National Laboratory, Upton, NY 11973, USA; d Research and Utilization Division, SPring-8/JASRI, 1-1-1 Kouto, Sayo, Hyogo 679-5198, Japan

**Keywords:** refractive lens, large aperture, hard X-ray focusing, momentum-resolved spectroscopy

## Abstract

One-dimensional kinoform and prism refractive lenses with large aperture and high transmittance at 22 keV have been investigated. A 12.0 µm focus size (full width at half-maximum) and an effective aperture of 0.85 mm, at a focal length of 705 mm and 21.747 keV, were achieved.

## Abstract 2

Large-aperture focusing lenses have been evaluated for momentum-resolved and flux-limited spectroscopy with hard X-rays.

## 1. Introduction   

X-ray focusing is important when performing experiments on small samples. This is especially true for flux-limited experiments, such as high-resolution spectroscopy. While high-efficiency solutions exist for focusing most of the beam from an insertion device at a third-generation source to a ∼100 µm spot, achieving a 10 µm-diameter focal spot without large losses is significantly more difficult.

The main issue for achieving small beam sizes at a third-generation source is focusing in the horizontal plane. Third-generation sources are very asymmetric, with a much smaller vertical source size and source divergence making vertical focusing by reflective, refractive or even crystal optics relatively straightforward. However, the order-of-magnitude lower brilliance in the horizontal direction results in a qualitatively different problem for the horizontal focus: the much larger source size means one must focus more strongly in the horizontal, compared with the vertical, to achieve a similar final beam size, while the larger source divergence in the horizontal (and larger source size) means that the acceptance of the focusing optic must be relatively large. In short, the numerical aperture of an optic for horizontal focusing must be much larger than for vertical focusing to achieve the same focused size without significant loss of intensity.

In the present paper we consider kinoform (Evans-Lutterodt *et al.*, 2003 ▶) and prism (Simon *et al.*, 2008 ▶, 2010 ▶) lenses as possible large-aperture focusing elements at 22 keV. These are both essentially refractive optics used in transmission, and, in principle, will suffer from absorptive losses. However, for the kinoform lenses the removal of much of the non-active material using lithographic techniques helps to increase transmission, while, for the prism lenses, the use of a low-*Z* material and a wave-guiding effect can improve its properties, in addition to the removal of the optically passive material.

We consider these lenses in the context of flux-limited momentum-resolved spectroscopic measurements, such as high-resolution inelastic X-ray scattering (Burkel, 2000 ▶; Sette *et al.*, 1998 ▶). Here one is concerned with the efficiency of the optic, defined as the ratio of photons in the focused spot to those of the incident beam, and its effect on the divergence of the beam, as well as the final focused beam size. In fact, for some experiments, one must be careful not to focus so strongly that the divergence of the incident beam degrades the momentum resolution. Therefore, it is desirable to have the possibility of relaxed focusing, *e.g.* a ∼100 µm beam size, with small divergence, and to have strong focusing, *i.e.* with a <∼10 µm beam size, and larger divergence for small samples where momentum resolution is not so crucial. Considering switching between these two cases, the ease of insertion of the optic into the beamline is also an issue. Here, the fact that the refractive optics we discuss do not change the direction of the beam makes them advantageous over reflection- or diffraction-based optics.

## 2. Lenses investigated   

### 2.1. Silicon kinoform lenses   

A kinoform lens (Evans-Lutterodt *et al.*, 2003 ▶) is a modified version of a compound refractive lens (Snigirev *et al.*, 1996 ▶), which is usually a metal with small drilled holes or pressed forms. The kinoform design allows removal of the passive material (contributing 2π phase-shifts) but retention of the lens shape, maintaining the focusing properties while significantly reducing absorption. Such a kinoform lens is a relatively low-loss and phase-preserving optic.

Silicon kinoform lenses were fabricated by deep reactive ion etching as discussed by Evans-Lutterodt *et al.* (2003 ▶). The procedure is (i) making a mask layer on a clean silicon wafer; (ii) spinning resist and baking; (iii) writing patterns on the resist with an electron-beam writer; (iv) developing; (v) transferring the patterns on the resist to the mask with a reactive ion etcher; (vi) removing the remaining resist; (vii) etching the silicon wafer patterned. Lithographic techniques with silicon are highly advanced, leading to the possibility of making fine structures and small focal spots.

**Figure 1:**
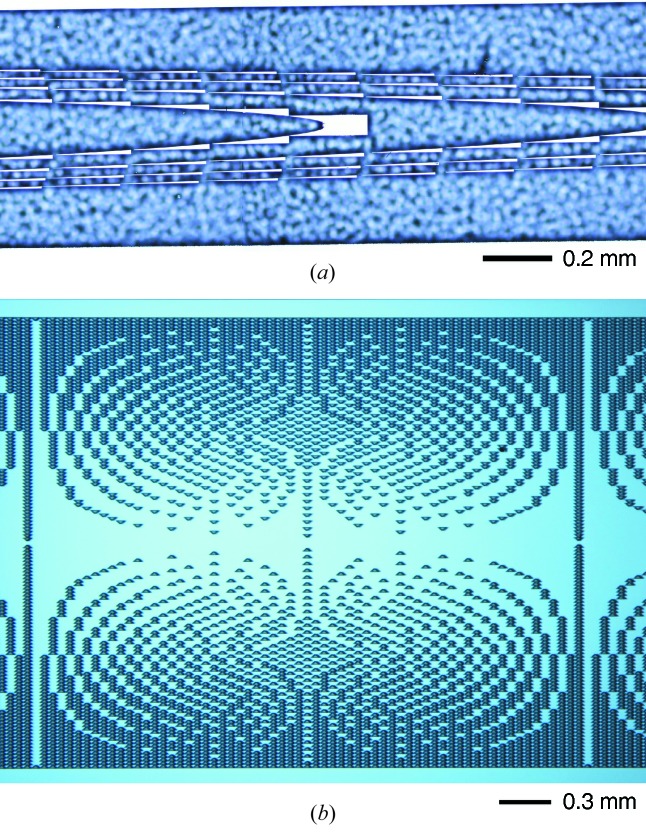
Photograph of sections of (*a*) the kinoform and (*b*) the prism lenses used in this work.

For apertures that are usefully large, the features near the extremum of the lens are difficult to fabricate. There are two strategies to overcome this problem. The first is to use multiples of 2π phase-shift, which makes all the features correspondingly larger. The second strategy is to use compound lenses consisting of an array of lenses, instead of single lenses. For example, we used three kinoform lenses: a single lens (short), a five-lens compound lens (medium) and a ten-lens compound lens (long). The key design parameters of the three different lenses are listed in Table 1 ▶. We prepared two sets of these three lenses, each of which was placed on a silicon wafer. Fig. 1(*a*) ▶ shows lens elements of the medium one.

### 2.2. SU-8 prism lenses   

A prism lens realises an effectively concave shape by repeating a highly regular shape (prism) (Jark *et al.*, 2004 ▶). These lenses consist of ∼10^4^ triangular prisms. At each prism the direction of the propagation is modified. In the prism lens used here, each prism is positioned on the curved path of the light through the lens for more effective focusing (Simon *et al.*, 2008 ▶). As the individual prism element deflects the beam by a fixed angle the prism arrays can be considered as waveguides and the focused beam size depends essentially on the size of the prisms.

The epoxy prism lenses were fabricated by deep X-ray lithography [see, for example, Wallrabe *et al.* (2008 ▶) for details]. Briefly, a negative tone absorber structure is fabricated on a mask. This mask is used to fabricate the lens in negative resist material. Electron-beam writing is used to fabricate an intermediate mask on a clean silicon wafer. The intermediate X-ray mask is copied into a working mask with higher absorber structure using soft X-ray lithography. Then the working mask is used to copy the lens structure in a SU-8 negative resist layer by deep X-ray lithography. Finally the exposed resist is developed and the lenses are ready to use.

We prepared three prism lenses on two silicon wafers. Fig. 1(*b*) ▶ shows lens elements of one of the prism lenses.

In total, we investigated six silicon kinoform lenses and three epoxy prism lenses. The effective height of the investigated lenses was measured to be >50 µm.

## 3. Method   

**Figure 2:**
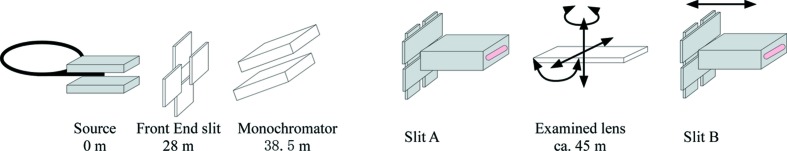
Schematic of the experimental set-up for lens evaluation. The lens was placed on a four-axis motorized stage. Slits A and B, with downstream ionization chambers, were motorized for motion perpendicular to the X-ray beam. The position of slit B along the X-ray beam was manually adjusted on an optical rail. A CCD camera was also sometimes mounted at the position of slit B.

Performance evaluation was carried out at BL35XU of SPring-8 (Baron *et al.*, 2000 ▶). A schematic of the optics set-up is shown in Fig. 2 ▶. The X-ray source was the third harmonic of the SPring-8 standard 32 mm-period undulator (Hara *et al.*, 1998 ▶). The source size is ∼0.650 mm in the horizontal. The beam was defined to 1.7 mm (H) × 0.3 mm (V) using a front-end slit at 28 m from the source. X-rays were monochromated to 21.747 keV by a liquid-nitrogen-cooled double silicon crystal monochromator (Mochizuki *et al.*, 2001 ▶) at 38.5 m from the source. The lenses were placed on a four-axis position control stage, at about 45 m from the source, between two slit-ionization chamber (S + IC) assemblages (Fig. 2 ▶). The incident and transmitted beam intensities were measured by the S + IC A and B assemblages, respectively. Each lens was roughly aligned by observing the transmitted (or focused) beam with a YAG-crystal/CCD-camera system. The optimal focal length was determined with an accuracy of better than 10 mm by moving slit B along the beam and finding the minimum beam size *via* horizontal scans of slit B. Lastly, the position and angle of the lens were precisely aligned by minimizing the beam size. The beam sizes were measured by scanning slit B (horizontal width <5 µm) or by scanning the edge of slit B. As there was no significant difference between the results of the slit scans and the edge scans, only the results of the slit scans will be shown in the next section.

## 4. Results   

### 4.1. Focusing properties   

A summary of the results is listed in Table 2 ▶. The focal lengths were 700–730 mm. The observed focal spot sizes, with slit A set at either 1 or 2 mm, were 11–15 µm FWHM, including an expected source contribution of about 10 µm.^1^

**Figure 3:**
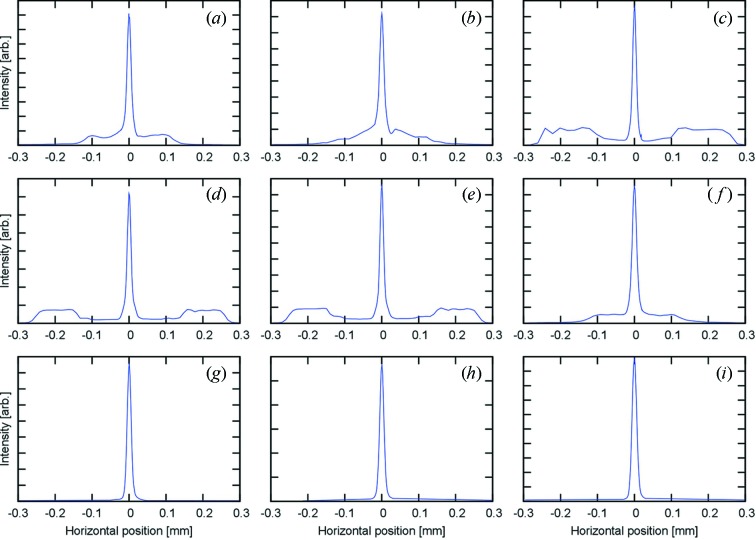
Focused line profiles measured with incident beam widths of 1.0 mm and 2.0 mm for the kinoform and prism lenses, respectively. Labels on the figures correspond to those shown in Table 2 ▶.

Focused beam profiles over a 600 µm range are shown in Fig. 3 ▶. Tails were intense and unrefracted beam was observed with some kinoform lenses. Some of the observed increase in transmission away from the focus can be explained by errors in the smaller lens features near the periphery. While the design and lithography is relatively straightforward to implement, the reactive ion etching of the patterns is somewhat less controlled. A key difficulty is that the etch undercut angle limits the etch depth of the lens and the uniformity of the lens properties along the depth. In the kinoform lenses fabricated here, the smallest features at the edge of the lens were completely undercut.

For the prism lenses, no unrefracted beam was observed, even with the 2.0 mm-width beam. However, since enlarging the incident beam size made the focal spot wider by ∼2 µm, the prism alignment might be slightly worse further from the optic axis of the lens.

We estimated the momentum resolution if these lenses are used in X-ray scattering measurements. If the incident beam has a Gaussian profile with 0.5 mm FWHM at the lens, the beam divergence is calculated to be less than 0.7 mrad (0.04°), including the effect of the lens aperture. This is acceptable for many experiments, being comparable with the mosaic spread of many crystals, and corresponding to, for a small-angle worst-case scenario, a blurring of momentum resolution of 0.154 nm^−1^ at 21.7 keV.

### 4.2. Aperture and efficiency   

**Figure 4:**
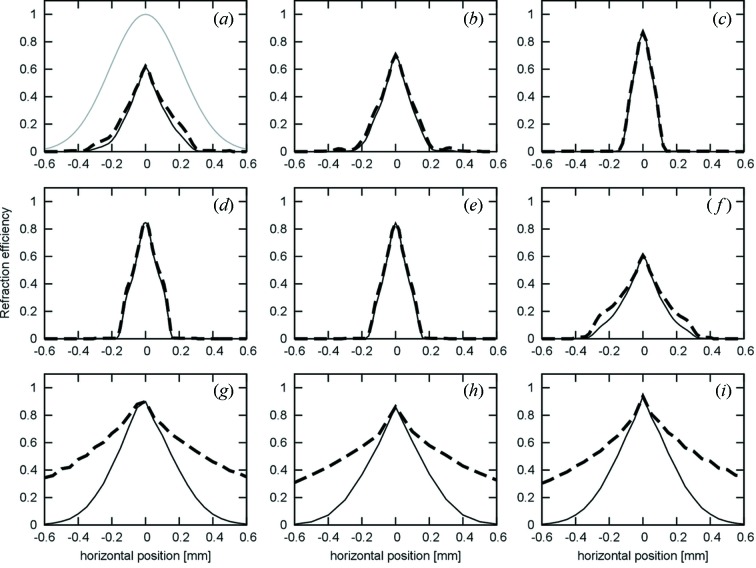
Aperture functions of the examined lenses (thick dotted line). Thin lines indicate calculated refraction efficiencies for a Gaussian incident beam with 0.5 mm FWHM [grey line shown only in (*a*)] into a 50 µm width at the optic axis of the lens. Labels on the figures correspond to those shown in Table 2 ▶.

The effective aperture of each lens was measured as follows. Slit A was reduced to a 0.05 mm width and fixed at the intense part of the X-ray beam from the source. Then, the lens and slit B (also 0.05 mm) were synchronously scanned relative to the incident beam so that slit B was always located at the expected focal point of the lens. The results for the kinoform lenses, Figs. 4(*a*)–4(*f*) ▶, indicate that the actual aperture, where X-rays can transmit to the focal line, was 0.7 mm at most. This is also related to the difficulty in the fabrication of lens features at the periphery. Photons close to the edge of the lens suffer greater absorption in passing through more material. We call the FWHM of the aperture function the ‘effective aperture’. The effective apertures were typically ∼0.2 mm and 0.7 mm for the kinoform and prism lenses, respectively, as seen in Fig. 4 ▶. The values are listed in Table 2 ▶. In the case of the prism lenses, the transmittance into the focal line was more than 20% at even 1.0 mm from the optic axis.

Using the aperture functions, we calculated the efficiency of each lens in a couple of different cases. We define this as 100 × [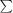 *f*_aperture_(*x*) × Int(*x*)]/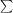
Int(*x*) (%), where *f*_aperture_ and Int are the aperture function of the lens and the intensity profile of the incident radiation, respectively, and *x* is a position perpendicular to the optic axis of the lens. If the intensity profile is a Gaussian function with a FWHM of 0.5 mm, the best efficiency for the kinoform lenses was 29% and that for the prism lenses was 69% (Fig. 4 ▶). In the case of a 1.0 mm FWHM, those for the kinoform and prism lenses are 17% and 56%, respectively.

## 5. Discussion   

The kinoform lenses achieved smaller beams sizes, and in fact they were probably limited not by the lens but by the effective source size of the beamline. Meanwhile, the SU-8 prism lenses achieved a larger effective aperture. The latter is consistent with an improved ratio of scattering to absorption for the lower-*Z* material in the prism lenses: the silicon scatters more, with a refractive index decrement, δ, of 1.02 × 10^−6^ *versus* 5.64 × 10^−7^ for the epoxy resin, but also absorbs much more, with an absorption coefficient of 7.53 cm^−1^ as compared with 0.49 cm^−1^ for the resin at 21.747 keV. Focusing on materials properties only, we expect the numerical aperture of a kinoform lens to scale with the ratio of scattering to absorption, like a conventional compound refractive lens. Therefore, neglecting fabrication issues, diamond lenses could have apertures almost an order of magnitude larger than the silicon lenses discussed here, and recent progress (Isakovic *et al.*, 2009 ▶) is somewhat encouraging. However, kinoform lenses are also fabrication-limited because the size of the structures becomes prohibitively small as one moves to larger apertures, further from the optic axis. The kinoform lenses, like a Fresnel zone plate, or any phase-preserving optic operating in transmission, must have 2π phase-shifts on a scale of the outer Fresnel zone size. Meanwhile, with their waveguide effect, the prism lenses allow high throughput but do not preserve phase. One can then consider combining these: a phase-preserving optic near to the optical axis, which then changes over into a fixed-feature-size wave-guiding optic when the proper phase structures become prohibitively small, beyond some distance from the axis. Such a mixed-method lens would allow either high-throughput operation or phase-preserving operation, depending on the size of the illuminating beam (or a slit in front of the optic), and, if made from diamond, could have an extremely large aperture.^2^

## 6. Conclusions   

This study shows that both the micro-fabricated refractive lenses can produce a ∼10 µm focused beam with relatively long focal lengths of 700 mm. The smallest focused spot size was achieved with one of the kinoform lenses, as is consistent with rather advanced processing techniques available for silicon. Owing to the current fabrication capabilities, the prism lenses have a definite advantage for the application discussed in this paper, where throughput is prioritized once a modest, ∼10 µm, focus is achieved. Given recent progress with diamond (Isakovic *et al.*, 2009 ▶), the throughput from the kinoform lens may increase.

## Figures and Tables

**Table 1:** Design parameters of the three types of kinoform lenses measured, consisting of a single-element lens and compound lenses consisting of arrays of five and ten lenses Increasing the number of lenses allows for larger apertures and improved resolution but comes at the price of insertion loss.

Lens type	Number of lenses in array	Individual lens focal length (m)	Aperture (mm)	Phase-shifts per lens element	Transmission of compound lens	Resolution of compound lens (nm)
Short	1	0.7	0.315	16π	0.84	250
Medium	5	3.5	0.780	8π	0.43	102
Long	10	7	1.1	8π	0.18	73

**Table 2:** Summary of the lens evaluation

	Silicon kinoform lens						SU-8 prism lens		
	Short	Medium	Long	Short	Medium	Long	No. 1	No. 2	No. 3
	(*a*)	(*b*)	(*c*)	(*d*)	(*e*)	(*f*)	(*g*)	(*h*)	(*i*)
Peak width (FWHM) (µm)	13.0†	14.3†	11.9†	11.6†	11.8†	14.7†	12.0‡	13.1‡	13.9‡
Focal length (mm)	724	724	724	723	724	726	705	729	729
Effective aperture§ (mm)	0.24	0.20	0.15	0.19	0.17	0.25	0.85	0.79	0.74

† The horizontal gap for slit A was 1.0 mm.

‡ The horizontal gap for slit A was 2.0 mm.

§ See text.

## Footnotes

1 Tests with the kinoform lenses suggested that a size of <6 µm could have been achieved without source size limits.

2 When the full aperture is used, the combination of coherently, phase-preserving, and incoherently transported beams might lead to complex fine structure within the focal spot.
